# Reproducing the Rift Valley fever virus mosquito-lamb-mosquito transmission cycle

**DOI:** 10.1038/s41598-020-79267-1

**Published:** 2021-01-14

**Authors:** Paul J. Wichgers Schreur, Rianka P. M. Vloet, Jet Kant, Lucien van Keulen, Jose L. Gonzales, Tessa M. Visser, Constantianus J. M. Koenraadt, Chantal B. F. Vogels, Jeroen Kortekaas

**Affiliations:** 1 Wageningen Bioveterinary Research, Lelystad, The Netherlands; 2 Laboratory of Entomology, Wageningen University & Research, Wageningen, The Netherlands; 3 Laboratory of Virology, Wageningen University & Research, Wageningen, The Netherlands; 4 Present Address: Department of Epidemiology of Microbial Diseases, Yale School of Public Health, New Haven, CT USA

**Keywords:** Biological techniques, Ecology, Microbiology, Diseases, Pathogenesis

## Abstract

Rift Valley fever virus (RVFV) is a mosquito-borne bunyavirus that is pathogenic to ruminants and humans. The virus is endemic to Africa and the Arabian Peninsula where outbreaks are characterized by abortion storms and mortality of newborns, particularly in sheep herds. Vector competence experiments in laboratory settings have suggested that over 50 mosquito species are capable of transmitting RVFV. Transmission of mosquito-borne viruses in the field is however influenced by numerous factors, including population densities, blood feeding behavior, extrinsic incubation period, longevity of vectors, and viremia levels in vertebrate hosts. Animal models to study these important aspects of RVFV transmission are currently lacking. In the present work, RVFV was transmitted to European (Texel-swifter cross-breed) lambs by laboratory-reared *Aedes aegypti* mosquitoes that were infected either by membrane feeding on a virus-spiked blood meal or by feeding on lambs that developed viremia after intravenous inoculation of RVFV. Feeding of mosquitoes on viremic lambs resulted in strikingly higher infection rates as compared to membrane feeding. Subsequent transmission of RVFV from lamb to lamb by infected mosquitoes was highly efficient in both models. The animal models described here can be used to study mosquito-mediated transmission of RVFV among the major natural target species and to evaluate the efficacy of vaccines against mosquito-mediated RVFV infection.

## Introduction

Rift Valley fever virus (RVFV) is a mosquito-borne RNA virus that causes severe disease in ruminants and humans^1^. The virus is transmitted via mosquitoes, predominantly of the genera *Aedes* and *Culex*^2^. Sheep are the most susceptible species, with disease being most severe in young lambs. RVFV has a tropism for the liver, with replication in hepatocytes resulting in focal to widespread hepatic necrosis^3,4^. In severe cases, hepatic necrosis is associated with bleeding tendencies, shock and death. In pregnant ewes, RVFV also targets maternal epithelial cells of the placenta and fetal trophoblasts, resulting in severe placental pathology and abortion^5^. Heavy mortalities among newborn lambs and abortion storms are key characteristics of RVF outbreaks. Infection of humans generally manifests as a self-resolving febrile illness, whereas a small percentage of patients develop encephalitis or hemorrhagic fever, the latter with high case fatality^3^. Although the risk of RVFV infection during human pregnancy is yet unclear, RVFV infections have been associated with miscarriage and recent studies have demonstrated that the virus replicates efficiently in human placental explants^5,6^. The threat that RVFV poses to human health is acknowledged by the World Health Organization that placed RVFV on the Blueprint list of priority diseases likely to cause future epidemics in the face of insufficient or absent countermeasures^7^.

Since its first description in the 1930s, RVFV has caused severe outbreaks on the African continent and the Arabian Peninsula, where the disease has remained enzootic and continues to cause epizootics and epidemics^1,4^. Interepizootic maintenance of RVFV is possibly explained by vertical transmission of the virus to the eggs of floodwater *Aedes* mosquitoes^8^. These species oviposit near shallow wetlands, known as dambos, which dry out during periods of drought and flood during heavy rains. These dambos provide ideal breeding grounds, as the eggs of floodwater mosquitoes depend on desiccation and rehydration for hatching. In desiccated form, the eggs can remain viable for long periods of time, possibly explaining how RVFV is maintained during periods of low or even apparent absent mosquito activity. Although this hypothesis is plausible, vertical transmission of RVFV to mosquito eggs has been demonstrated only once, and in only one mosquito species, namely *Aedes* (*Neomelaniconion*) *mcintoshi* Huang, a species cited before 1985 as *Ae. lineatopennis*^8^. Additionally, evidence is accumulating that RVFV is maintained in mosquito-ruminant-mosquito cycles in the absence of outbreaks^9,10^.

Apart from re-emergence of RVFV in enzootic areas, climate change, globalization, increasing populations of susceptible animals, and changing ecologies of mosquito vectors may facilitate future incursions into currently unaffected areas. This concern is fueled by data that suggest that over 50 mosquito species, many of which with a global distribution, can potentially act as vectors of RVFV^2^. These data are based on isolation of RVFV from field-collected mosquitoes and from vector competence experiments in laboratory settings, in which the virus is taken up by mosquitoes by membrane feeding on a RVFV-spiked blood meal^2,11,12^. However, to more firmly establish the ability of a mosquito species to act as a vector, experiments are required in which the virus is transmitted among susceptible animals via infected mosquitoes. In such experiments, factors such as mosquito population density, blood feeding behavior, and longevity can be taken into account to determine the vectorial capacity of a mosquito vector^13^. Furthermore, such experiments could address the influence of viremia levels in different vertebrate hosts on mosquito-mediated transmission.

In the present work, *Aedes aegypti* mosquitoes were infected with RVFV after membrane feeding on a virus-spiked blood meal or after feeding on lambs that developed viremia after intravenous inoculation of RVFV. The virus was subsequently transmitted by these mosquitoes to naive lambs. The presented animal models can be used to study environmental, vector and host factors that contribute to RVFV transmission and to determine the efficacy of vaccines against natural, mosquito-mediated infection.

## Results

### Infection of lambs via intravenous inoculation of RVFV followed by transmission of the virus between lambs and mosquitoes

**Figure 1:**
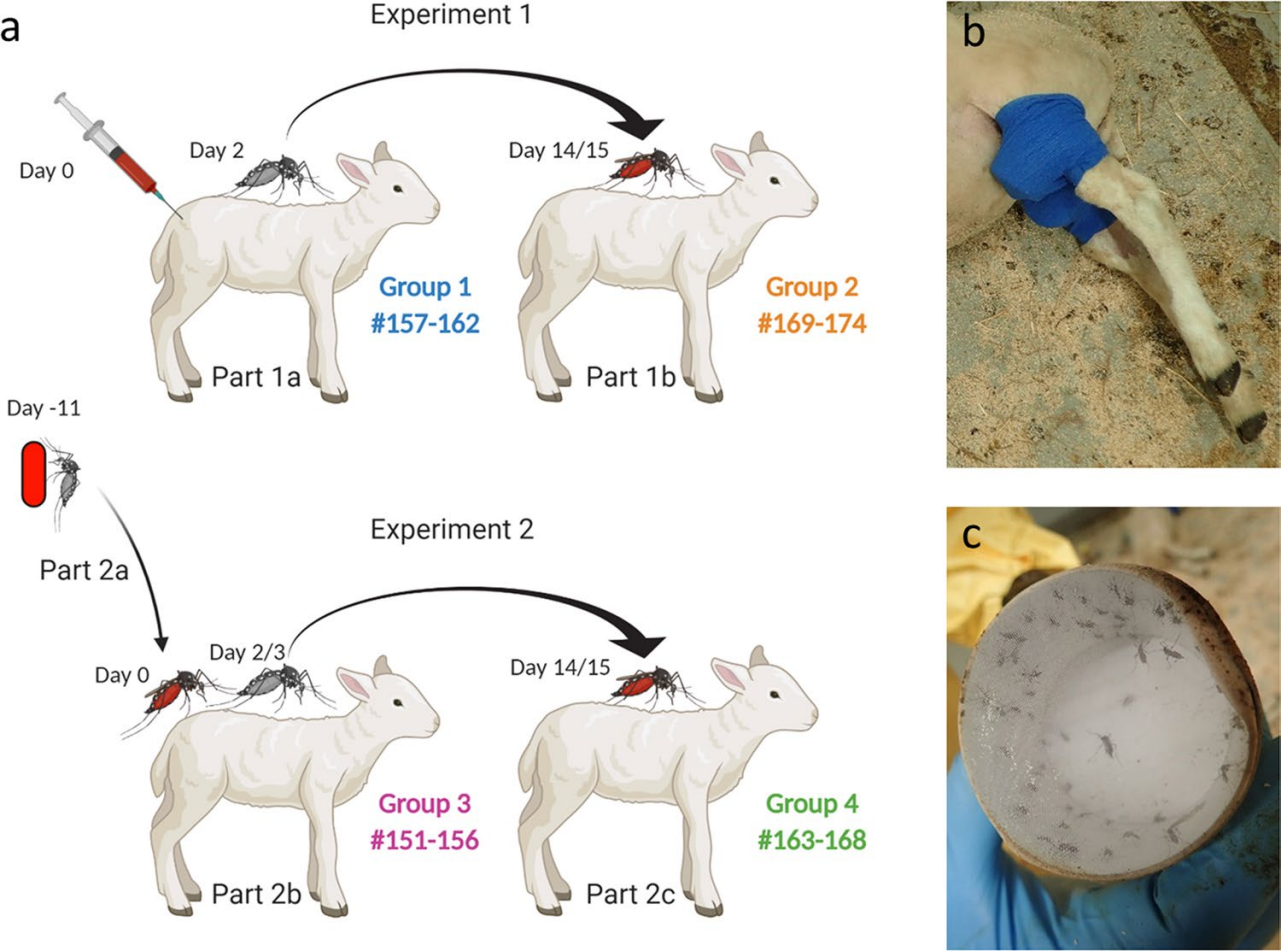
Schematic representation of two experiments performed to study transmission of RVFV between mosquitoes and lambs. (**a**) In Experiment 1, lambs were inoculated via IV route with RVFV. Two days later, when pyrexia was noted, naive mosquitoes were allowed to take a blood meal from the lambs (Part 1a). Fully engorged mosquitoes were incubated for 12–13 days, after which they were allowed to take a blood meal from lambs of Group 2 (Part 1b). Before start of a parallel experiment (Experiment 2), mosquitoes were allowed to feed on a virus-spiked blood meal using a Hemotek apparatus (Part 2a). Fully engorged mosquitoes were incubated for 11 days, until they were allowed to take a second blood meal on lambs of Group 3 (Part 2b). Two to three days later, when pyrexia was measured, naive mosquitoes were allowed to take a blood meal from the lambs of Group 3 (Part 2b). Fully engorged mosquitoes were incubated for 12–13 days, after which the mosquitoes were allowed to take a second blood meal from naive lambs of Group 4 (Part 2c). The presence of RVFV in mosquito saliva samples was determined retrospectively using forced salivation and virus isolation on Vero cells. (**b**) Representative picture of a cardboard box containing mosquitoes placed on the hind legs of sedated lambs. (**c**) Representative picture of a cardboard box after mosquito feeding. Experimental details according to the ARRIVE guidelines^30^ are presented in Supplementary Table S1 online.

**Figure 2:**
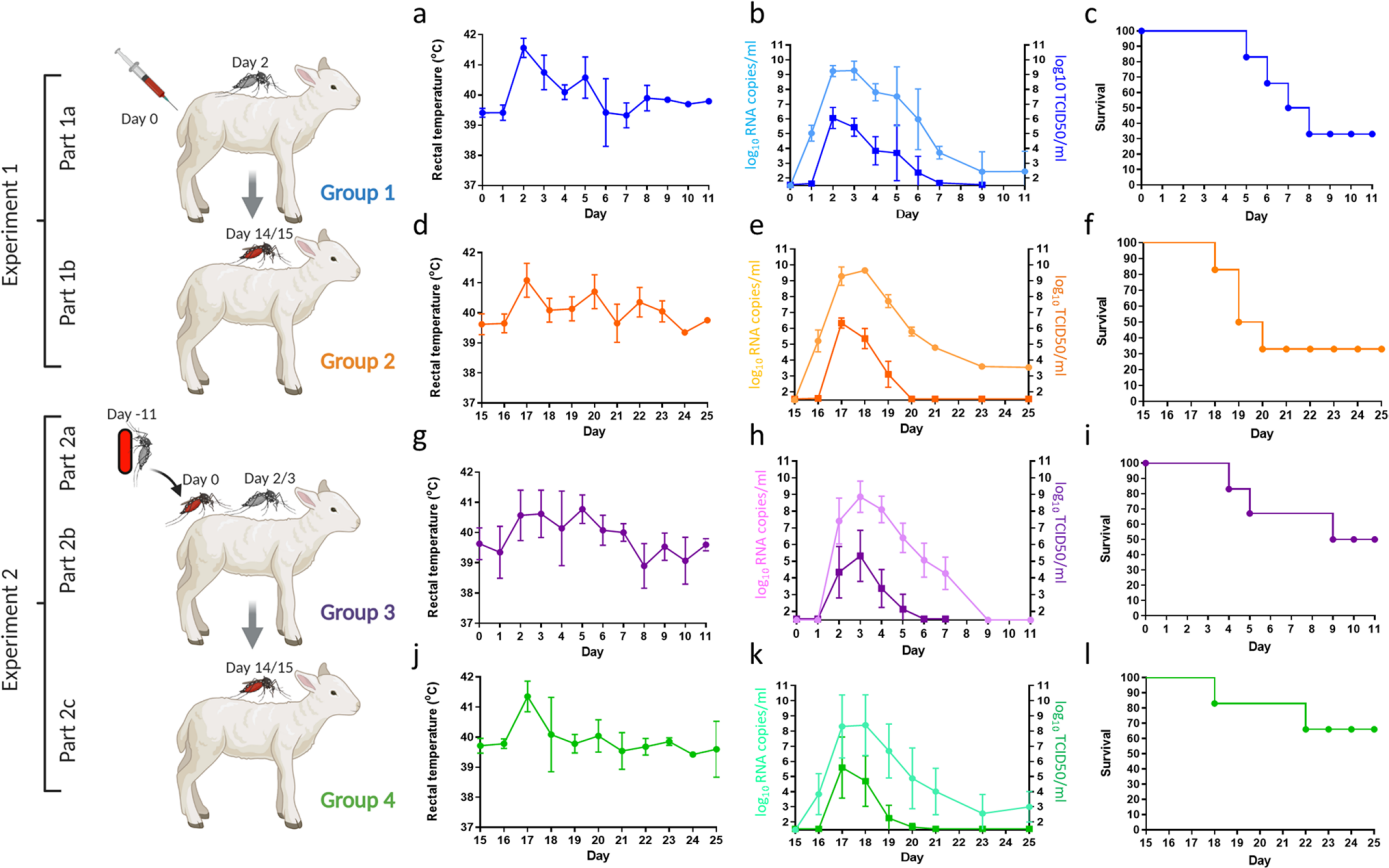
Analysis of lambs exposed to RVFV via intravenous injection or via infected mosquitoes. Lambs (n = 6/group) were exposed to RVFV either via intravenous inoculation (Group 1) or via infected mosquitoes (Groups 2, 3 and 4), as schematically presented at the left. Mosquitoes that fed on lambs of Group 1 on day 2 were used to transmit the virus to lambs of Group 2 on day 14 or 15. Lambs of Group 3 were exposed to mosquitoes that membrane fed on a RVFV-spiked blood meal 11 days earlier. Naive mosquitoes were allowed to take a blood meal from these lambs on day 2 or 3. After an incubation period, these mosquitoes were used to transmit the virus to lambs of Group 4 on day 14 or 15. Rectal temperatures (**a**,**d**,**g**,**j**), viral RNA and infectious virus in plasma samples (**b**,**e**,**h**,**k**) and survival rates (**c**,**f**,**i**,**l**) are presented. Error bars represent SDs.

**Figure 3:**
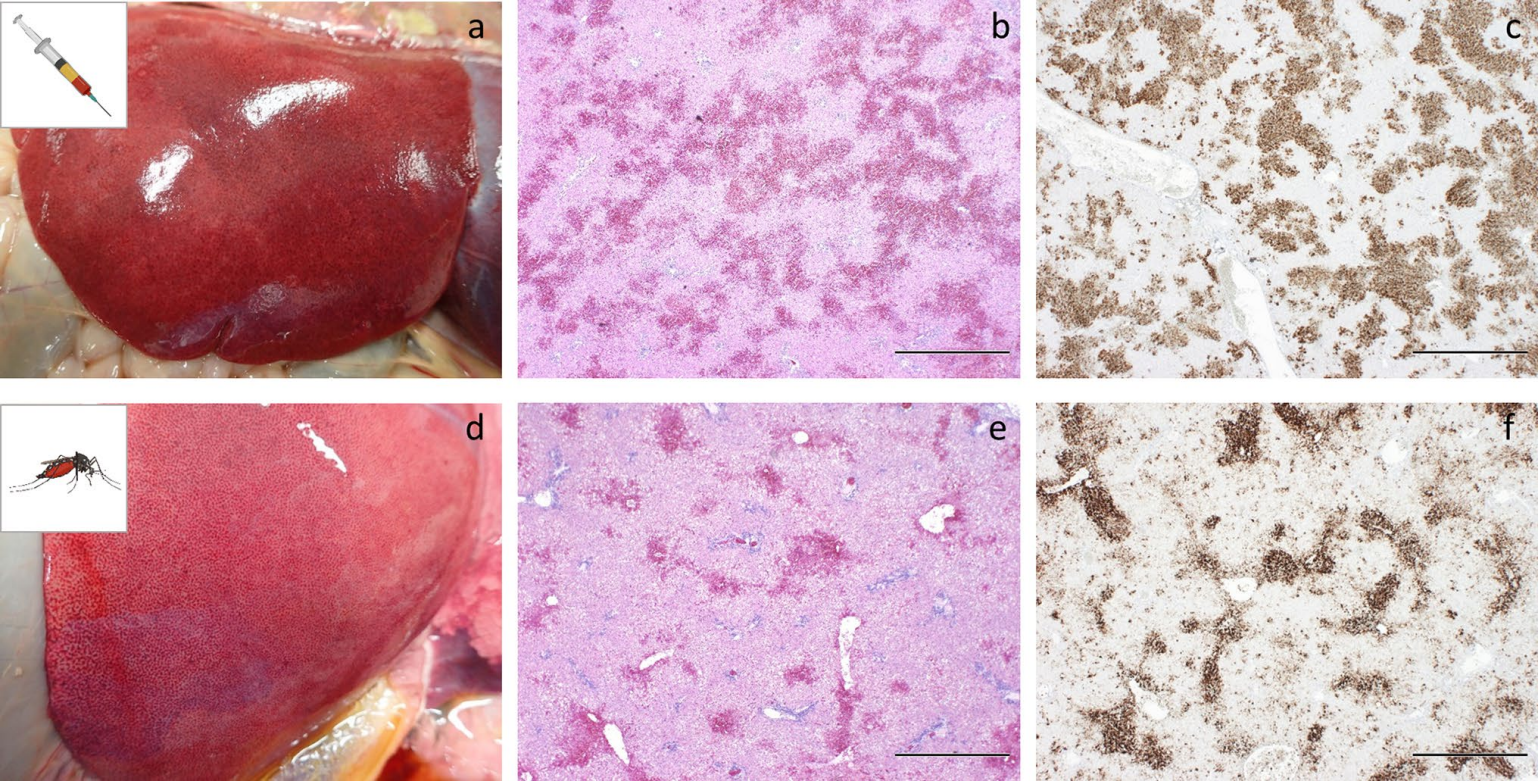
Representative pathology in lambs exposed to RVFV via IV-inoculation or infected mosquitoes. Panels **a**–**c** were obtained from lamb #161 (IV inoculation). Panels **d**–**f** were obtained from lamb #154 (mosquito exposure). Both lambs were euthanized after reaching a HEP. Swollen liver with mottled appearance indicative of hepatic degeneration/necrosis (**a**,**d**), H&E staining of liver sections showing multifocal to bridging necrosis of hepatocytes with haemorrhages (**b**,**e**). IHC staining of a liver section with mAb 4-D4 showing strong immunolabelling for RVF antigen of the areas with degeneration and necrosis of hepatocytes (**c**,**f**). Bar = 1,000 μm.

A group of 6 lambs (Group 1, #157–162) was intravenously (IV) inoculated with RVFV on day 0 (Fig. 1a, Experiment 1). Upon onset of pyrexia, previously shown to correspond with peak viremia^14^, mosquitoes were allowed to take a blood meal. To this end, mosquitoes were placed on sedated lambs in cardboard containers, bound to each of the hind legs with elastic tape (Fig. 1b,c). The average mosquito feeding rate was 85% (± 7%) (Table 1). Fully engorged mosquitoes were collected and maintained in an insect incubator as separate groups per lamb. All IV inoculated lambs developed pyrexia (Fig. 2a), became listless and were disinclined to feed from day 2 onwards. Analysis of daily collected plasma samples with reverse-transcriptase quantitative PCR (RT-qPCR) and virus isolation demonstrated that all lambs developed high viremia, which peaked on day 2 post inoculation (Fig. 2b), confirming that mosquito feeding occurred at peak viremia. One lamb (#159) acutely succumbed to the infection on day 4, one lamb acutely succumbed on day 5 (#157), one lamb had to be euthanized after reaching a humane end-point (HEP) on day 6 (#161) and one on day 7 (#162), resulting in a case fatality rate (CFR) of 67% (Fig. 2c). Necropsies of fatal cases revealed typical pathological manifestations of RVFV infection, with focal to widespread hepatic necrosis (Fig. 3a–c).

**Table 1:** Quantification of transmission parameters.

Experiment 1, Part 1a					Experiment 1, Part 1b									
Group 1#:	Viremia day 2 (log10)		Naive mosquitoes		Group 2#:	Infected mosquitoes			Viremia Day 17 (log10)					
	TCID_50_/ml	RNA copies	Fed/total (%fed)	Survivors/total (%)		fed/total (% fed)	SP/tested (%SP)	ESP/fed (%ESP)	TCID_50_/ml	RNA copies/ml				
157	5.1	8.7	70/89 (79)	63/70 (90)	169	51/63 (81)	13/44 (30)	15/51 (30)	6.2	9.8				
158	6.5	9.2	86/97 (89)	82/86 (95)	170	73/82 (89)	19/60 (32)	23/73 (32)	6.5	9.8				
159	6.7	9.7	93/98 (95)	86/93 (92)	171	59/86 (69)	23/53 (43)	26/59 (44)	6.2	9.4				
160	5.3	8.8	80/95 (84)	72/80 (90)	172	67/72 (93)	22/57 (39)	26/67 (39)	6.9	9.3				
161	6.2	9.6	70/92 (76)	51/70 (73)	173	31/51 (61)	14/28 (50)	16/31 (52)	6.2	9.6				
162	6.7	9.7	85/94 (90)	75/85 (88)	174	47/75 (63)	13/45 (29)	14/47 (30)	6.0	8.3				
Average	6.1 ± 0.7	9.2 ± 0.5	85 ± 7%	88 ± 8%	Average	76 ± 14%	37 ± 9%	38 ± 9%	6.3 ± 0.3	9.4 ± 0.6				

Experiment 2, Part 2a				Experiment 2, Part 2b					Experiment 2, Part 2c					
Group 3#:	Infected mosquitoes			Group 3#:	Viremia day 2/3 (log10)		Naive mosquitoes		Group 4#:	Infected mosquitoes			Viremia Day 17 (log10)	
	Fed/total (%fed)	SP/tested (%SP)	ESP/fed (%ESP)		TCID_50_/ml	RNA copies	fed/total (% fed)	Survivors/total (%)		fed/total (%fed)	SP/tested (%SP)	ESP/fed (%ESP)	TCID_50_/ml	RNA copies/ml
151	64/92 (70)	0/0 (0)^a^	N.A	151	4.4	7.9	85/102 (83)	75/85 (88)	163	64/75 (85)	21/53 (40)	25/64 (39)	6.9	9.7
152	35/96 (36)	1/21 (5)	2/35 (6)	152	5.1	8.6	87/100 (87)	75/87 (86)	164	54/75 (72)	25/48 (52)	28/54 (52)	6.5	9.1
153	81/101(80)	5/54 (9)	8/81 (10)	152^b^	5.5	9.1	96/100 (96)	73/96 (76)	168	55/73 (75)	6/46 (13)	7/55 (13)	6.2	8.5
154	73/99 (74)	3/54 (6)	4/73 (6)	153	2.5	7.0	86/94 (91)	81/86 (94)	165	66/81 (81)	1/61 (2)	1/66 (2)	< 1,6	4.2
155	45/102(44)	2/37 (5)	2/45 (4)	154	6.7	9.4	96/102 (94)	88/96 (92)	166	78/88 (89)	16/65 (25)	19/78 (24)	6.7	9.6
156	72/90 (80)	7/41 (17)	12/72(17)	155	5.8	8.4	87/100 (87)	67/87 (77)	167	57/67 (85)	16/49 (33)	19/57 (33)	5.8	8.7
Average	64 ± 19%	8 ± 5%	9 ± 5%	Average	5.0 ± 1.4	8.4 ± 0.9	90 ± 5%	86 ± 8%	Average	81 ± 6%	27 ± 18%	27 ± 18%	6.4 ± 0.4^c^	8.3 ± 2

In Experiment 1 (Part 1a), lambs from Group 1 (n = 6, blue) were IV-inoculated with RVFV on day 0. When pyrexia was measured on day 2, *Ae. aegypti* mosquitoes were allowed to take a blood meal. Viremia levels on this day were determined by RT-qPCR and virus isolation using plasma samples. In Experiment 2 (Part 2a), mosquitoes were infected via membrane feeding on a RVFV-spiked blood meal. After 11 days incubation, the mosquitoes were allowed to feed on lambs of Group 3 (day 0), after which the presence of RVFV in mosquito saliva samples was determined. When pyrexia in lambs of Groups 1 and 3 was noted on day 2 or 3, naive mosquitoes were placed on the lambs and viremia levels on this day were determined by RT-qPCR and virus isolation using plasma samples. The total numbers of engorged mosquitoes (fed) and the total numbers of mosquitoes used for feeding (total) are indicated, as well as the percentages fed (%fed). Engorged mosquitoes were maintained in an insect incubator for 12–13 days. After this incubation period, surviving mosquitoes were allowed to feed on naive lambs (Groups 2 and 4). The presence of RVFV in mosquito saliva samples was determined by virus isolation on Vero cells after feeding on the lambs. Arrows indicate the lambs between which RVFV was transmitted. The numbers of saliva-positive mosquitoes are indicated (SP) as well as the numbers of mosquitoes tested (tested) and the percentage of saliva-positive samples (%SP). Because not all mosquitoes survived the incubation period, the expected number of saliva positives (ESP) was extrapolated by multiplying the %SP x fed. Titers of infectious virus and RNA copies in the blood of the lambs from Groups 2 and 4 at peak viremia (experimental day 17) are also indicated. N.A., not applicable.

^a^This lamb recovered early from sedation and violently responded to the placement of the cardboard box, resulting in mortality of all blood-fed mosquitoes.

^b^Lamb #156 was not included in Experiment 2, Part b, as this lamb succumbed to the infection before mosquitoes could feed. The corresponding mosquitoes were allowed to feed on lamb #152 on day 3.

^c^No infectious virus was detected in plasma of lamb #165. This sample was therefore not included when calculating the average viremia level.

The average survival rate of blood-fed mosquitoes after 13 days of incubation was 88% (± 8%) (Table 1). Following this incubation period, the 6 groups of surviving mosquitoes were each placed on naive lambs (Group 2, #169–174, Fig. 1a) (on average 72 mosquitoes per lamb) and allowed to take a second blood meal as described above. The average feeding rate of these mosquitoes was 76% (± 14%) (Table 1). Two days post feeding (dpf) on the naive lambs (day 17), all lambs developed high fever (Fig. 2d). One lamb (#173) reached a HEP and was euthanized on day 18 (dpf 3), two lambs (#169 and #174) succumbed acutely on day 19 (dpf 4) and one (#170) on day 20 (dpf 5), resulting in a CFR of 67% (Fig. 2f). Similar as observed in Group 1, all lambs that succumbed to the infection revealed typical signs of RVFV infection at necropsy. Analysis of plasma samples by RT-qPCR and virus isolation confirmed that peak viremia occurred on 2 dpf (day 17) (Fig. 2e). As all mosquito-exposed lambs developed viremia, transmission of RVFV by *Ae. aegypti* mosquitoes from viremic lambs to naive lambs was deemed very efficient.

To retrospectively assess the number of RVFV-positive mosquitoes that had fed on each naive lamb, mosquito saliva samples were collected by forced salivation and assayed for the presence of virus. These analyses demonstrated that 38% (± 9%) of the mosquitoes contained RVFV in saliva and suggested that each of the lambs from Group 2 were exposed to a minimum of 14 and a maximum of 26 saliva-positive mosquitoes (Table 1).

Between group comparisons of the experimental outcomes showed no significant differences (P < 0.05) in onset, viremia levels and peak of viremia between IV-inoculated lambs (Group 1) and lambs exposed to 14–26 RVFV-positive *Ae. aegypti* mosquitoes (Group 2). Similar morbidity and mortality rates were also observed (Fig. 2c,f and Table 1). In addition, no differences in mean viremia (AUC and peak viremia) were observed, neither in the variance of the measured variables (Fig. 2 and Supplementary Table S2 online). Given these results, mosquito-mediated transmission of RVFV from IV-inoculated lambs to naive lambs was deemed efficient and robust, with all exposed animals developing viremia and clinical signs with low variability observed in measured outcome parameters.

### Infection of mosquitoes via membrane feeding on a RVFV-spiked blood meal, followed by transmission of the virus between mosquitoes and lambs

In a parallel experiment, we investigated how efficiently RVFV can be transmitted between mosquitoes and lambs, starting with mosquitoes that ingested the virus via membrane feeding (Fig. 1a, Experiment 2). To this end, mosquitoes were fed on a bovine erythrocyte suspension containing 10^7.5^ TCID_50_/ml of RVFV, using a Hemotek membrane feeder. After 11 days incubation, the mosquitoes were allowed to feed on lambs from Group 3 (lambs #151–156) (on average 97 mosquitoes per lamb). Feeding was overall successful, although lamb #151 recovered early from its sedation, and violently responded to the cardboard boxes attached to its legs, resulting in death of all (already engorged) mosquitoes. Virtually all other mosquitoes survived the feeding period with an average feeding rate of 64% (± 19%) (Table 1). Retrospectively, forced salivation analysis showed that 9% (± 5%) of the mosquitoes used to infect lambs of Group 3 contained RVFV in saliva, which suggested that each lamb was exposed to 2–12 saliva-positive mosquitoes.

All mosquito-exposed lambs of Group 3 manifested with pyrexia (Fig. 2g) and analysis of plasma samples by RT-qPCR and virus isolation demonstrated that viremia peaked on days 2 or 3 (Fig. 2h and Table 1). All lambs developed clinical signs including lethargy and reduced appetite. One lamb (#156) succumbed acutely to the infection on day 3 and another lamb (#154) had to be euthanized upon reaching a HEP on day 4. Lamb #151 reached a HEP on day 8, resulting in a CFR of 50% (Fig. 2i). Necropsies of euthanized lambs revealed focal to widespread hepatic necrosis associated with RVFV antigen (Fig. 3d–f).

In line with experiment 1, naive mosquitos were placed on lambs of Group 3 when pyrexia was noted. Considering that not all lambs developed pyrexia on day 2, some of the lambs were exposed to mosquitoes on day 3 (Table 1). Of note, lamb #156 succumbed acutely before naive mosquitoes could be allowed to feed on this animal. The mosquitoes that were meant to feed on this lamb were instead allowed to feed on lamb #152 on day 3 (note that another group of mosquitoes fed on the same lamb on day 2). Between 85 and 96 naive mosquitoes were used for feeding and the average feeding rate was 90% (± 5%). Of the fully engorged mosquitoes (fed on Group 3), 86% survived the incubation period of 12–13 days. Surviving mosquitoes were subsequently placed on the naive lambs from Group 4 (lambs #163–168) (average 77 mosquitoes per lamb). Forced salivation analysis showed that 27% (± 18%) of the mosquitoes contained RVFV in saliva, which suggested that lambs of Group 4 were each exposed to 1–28 saliva-positive mosquitoes (Table 1).

All lambs from Group 4 developed pyrexia 2 days post mosquito exposure, corresponding to study day 17 (Fig. 2j). Analysis of plasma samples by RT-qPCR and virus isolation demonstrated that peak viremia occurred on the same day (Fig. 2k). Lamb #163 reached a HEP and was euthanized 3 days post feeding, corresponding to day 18. Lamb #164 reached a HEP 7 days post feeding, corresponding to day 22, resulting in a CFR of 33% (Fig. 2l).

Interestingly, one lamb from Group 3 (#153), revealed relatively low viral RNA levels in plasma samples and titers of infectious virus just above the detection limit of the assay (10^2.5^ TCID_50_/ml on day 3). Retrospective analysis suggested that this lamb was exposed to 8 membrane-fed RVFV saliva-positive mosquitoes (Table 1). Despite very low level of infectious virus in the blood of lamb #153, RVFV was transmitted successfully to lamb #165 of Group 4. Whereas lamb #165 developed detectable levels of viral RNA, no infectious virus was isolated (Table 1). Analysis of mosquitoes used to transmit virus from lamb #153 to #165 suggested that lamb #165 was exposed to one infected mosquito. Both lambs, #153 and #165 did not develop clinical signs and it was also interesting to find that all lambs, including #153 and #165, developed anti-nucleocapsid antibodies, as determined by ELISA, as well as neutralizing antibodies, which are known to correlate with protection (see Supplementary Fig. S1 online).

No significant differences (P < 0.05) in peak viremia or overall viremia during the course of infection were observed between Groups 3 and 4 . Variances were also similar, however the presence of sheep #153 and #165 in each of these groups led to high variances in the measured variables (Supplementary Table S2 online).

### Comparing RVFV transmission to mosquitoes after membrane feeding on a virus-spiked blood meal or on viremic lambs

**Figure 4:**
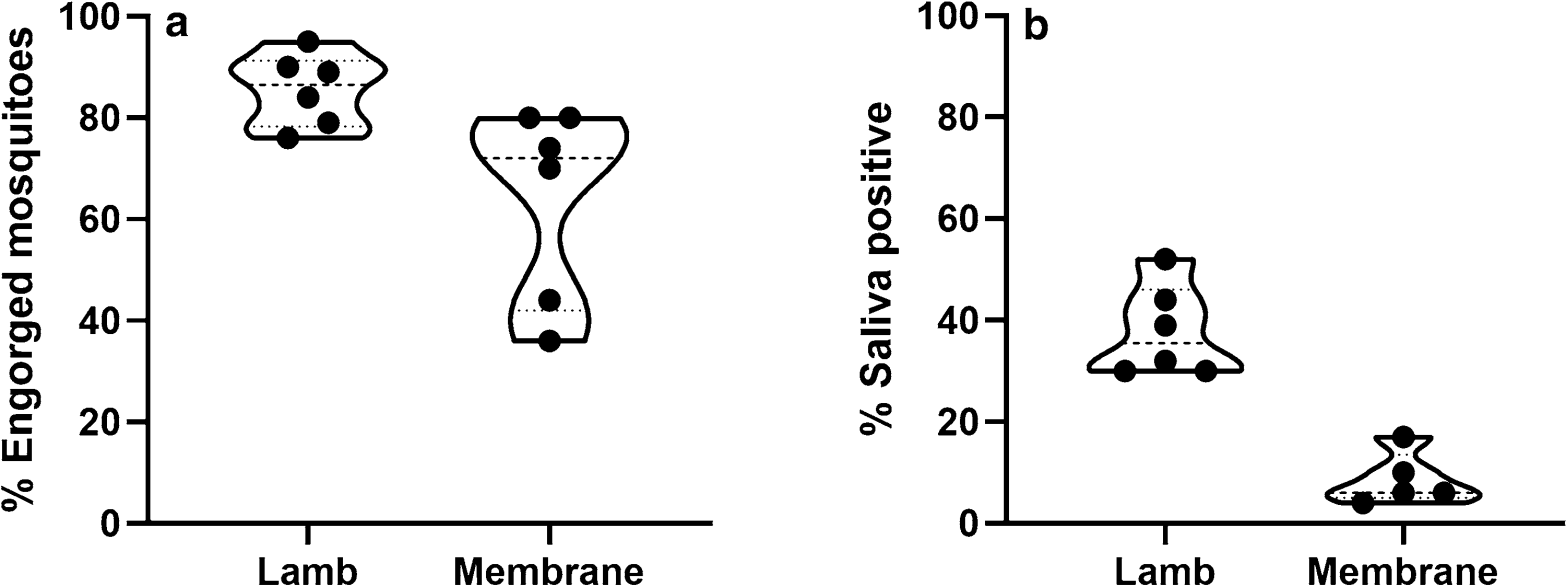
Feeding rates depicted as % engorged mosquitoes (**a**) and percentages of RVFV saliva-positive mosquitoes (**b**) after feeding on a viremic lamb or after membrane feeding on a virus-spiked blood meal. Mosquitoes were fed on a viremic lamb in Experiment 1, part 1a, or via membrane in Experiment 2, part 2a. Percentages of RVFV saliva-positive mosquitoes were determined after an incubation period of 15–21 days (Experiment 1), or after 14–21 days (Experiment 2). Virus in saliva was demonstrated by incubation on Vero cells, followed by scoring of CPE. Data are presented in violin plots with median and quartiles. Dots represent individual measurements.

In the present work, two methods were used to obtain mosquitoes that contained RVFV in saliva. Mosquitoes were fed either on viremic lambs or membrane-fed on a virus-spiked blood meal. The feeding rate (mean ± SD) in mosquitoes fed on needle-inoculated, viremic lambs (85% ± 7) was significantly higher (p = 0.002) than feeding rates of mosquitoes fed on a virus-spiked blood meal (64% ± 19). The percentage of saliva-positive (surviving) mosquitoes was also much higher (p < 0.001) when mosquitoes were fed on viremic lambs (38% ± 9%) as compared to membrane feeding (9% ± 5%) (Fig. 4), whereas the average virus titer during mosquito feeding on the lambs was 30-fold lower.

## Discussion

We here report the reproduction of the complete RVFV transmission cycle under controlled conditions. The established animal models can be used to study transmission of RVFV between laboratory-reared *Ae. aegypti* mosquitoes and lambs either initiating with IV inoculation of lambs, or with mosquito feeding on a RVFV-spiked blood meal. Although both models result in efficient transmission of the virus among lambs, our results show that feeding on viremic lambs results in much higher percentages of RVFV saliva-positive mosquitoes as compared to membrane feeding. Our results thereby underscore the previous notion that membrane feeding may result in a significant underestimation of vector competence and stress the value of experiments involving vertebrate hosts^15,16^.

Apart from evaluating the two models individually, our experiments also enabled us to directly compare the outcome of infection following IV inoculation or exposure to infected mosquitoes. Specifically, in Experiment 1, lambs were IV inoculated with a dose of 10^5^ TCID_50_ (Group 1) whereas in Experiment 2, lambs were exposed to infected mosquitoes (Group 3), which were fed earlier on a RVFV-spiked blood meal. Retrospective analyses of mosquito saliva suggested that these lambs were exposed to an average of 9 RVFV saliva-positive mosquitoes. This resulted (although not statistically significant) in an average lower peak rectal temperature, later onset and lower level of viremia, and a lower mortality rate when compared to the lambs inoculated via IV route with a dose of 10^5^ TCID_50_. Mosquitoes that were fed on the viremic lambs from both groups were subsequently allowed to feed on naive lambs from Groups 2 and 4. With one notable exception, all lambs developed pyrexia and viremia. The exception was lamb #165 from Group 4, which had an RNA level of 10^4.2^ copies/ml at dpf 2, which is below the limit of detection of our virus isolation assay. Interestingly, this lamb was exposed to mosquitoes that had earlier fed on lamb #153, which was an outlier itself from Group 3, displaying a low viremia level (10^2.5^ TCID_50_/ml) at the moment of mosquito feeding, possibly explaining the low number of mosquitoes that became infected after feeding on this lamb. Specifically, lamb #165 seemed to be exposed to only one saliva-positive mosquito, developing very low viremia after this exposure. The other lambs in both groups (Groups 2 and 4) were exposed to an average of 20 saliva-positive mosquitoes and the virological and clinical outcomes in these groups were highly similar to those resulting from IV inoculation (Group 1).

Intravenous inoculation of 10^5^ TCID_50_ of RVFV is currently applied in our laboratory to evaluate efficacy of vaccines which, according to the present study, seems to result in virological and clinical outcomes that mimic those following exposure to ~ 20 RVFV saliva-positive mosquitoes. It is interesting to consider if exposure to 20 RVFV saliva-positive mosquitoes is representative to the field situation. An entomological study performed during the Kenyan outbreak of 2006/2007, revealed an average infection rate below 3 per 1000 mosquitoes^17^. Although depending on vector densities and attack rates, this finding suggests that animals are unlikely to be exposed to RVFV via more than one mosquito in the field at any given moment, even during outbreaks. It would thus be valuable to evaluate virological and clinical outcome after exposure to a single saliva-positive mosquito in future experiments.

The efficient transmission of RVFV from lamb to lamb in the present work was facilitated by the use of laboratory-reared *Ae. aegypti* mosquitoes. We selected these mosquitoes for our studies as they are easily reared to very high numbers and as they take multiple bloodmeals in a single gonotrophic cycle^18^. It must be noted, however, that RVFV was never detected in *Ae. aegypti* collected from the field. This can be explained by differences in susceptibility between laboratory-reared mosquitoes and field-collected mosquitoes. Alternatively or additionally, the lack of detection of RVFV in field-collected *Ae. aegypti* mosquitoes may be explained by host preference. Having a preference for human blood, *Ae. aegypti* is mostly found in urban areas, whereas RVFV is most prevalent in rural areas where ruminants reside^19^. Nevertheless, the extremely efficient transmission of RVFV as determined in the present work should be considered a warning. *Ae. aegypti* is the primary vector of four arboviruses with major impact on human health: chikungunya virus, dengue virus, yellow fever virus, and Zika virus and growing populations of humans and livestock will facilitate encounters of *Ae. aegypti* and RVFV. Therefore, further studies are required to evaluate the risk of human-to-human transmission of RVFV by *Ae. aegypti* mosquitoes, which could result in urban transmission cycles. Considering this, it is important to determine if viremia levels in infected humans are sufficient to facilitate urban transmission cycles. One study, in which viremia levels approaching 10^6^ TCID_50_/ml were recorded suggests that humans may not be a dead-end host for RVFV as currently assumed^20^.

In conclusion, our work has demonstrated that RVFV can be transmitted efficiently from lamb to lamb via laboratory-reared *Ae. aegypti* mosquitoes and thereby provides novel methods to investigate environmental, vector and vertebrate host factors that affect transmission of RVFV in the field. Importantly, the methods can be used to evaluate efficacy of vaccines against mosquito-mediated exposure.

## Methods

### Virus and cells

RVFV strain 35/74 was originally isolated from the liver of a sheep that died during a RVFV outbreak in the Free State province of South Africa in 1974^21^. The strain was previously passaged four times in suckling mouse brain and three times in BHK cells. The virus used for IV inoculation of sheep was prepared by a further amplification in BHK-21 cells (ATCC CCL-10) cultured in CO_2_-independent medium (CIM, Invitrogen), supplemented with 5% FBS (Bodinco) and 1% Pen/Strep (Invitrogen).

To prepare a virus-spiked blood meal for membrane feeding of mosquitoes, the virus was amplified in *Aedes albopictus* C6/36 cells (ATCC CRL-1660). To this end, C6/36 cells were inoculated with a multiplicity of infection of 0.005 and cultured at 28 °C in absence of CO_2_ in L-15 medium (Sigma) supplemented with 10% fetal bovine serum (FBS), 2% Tryptose Phosphate Broth (TPB) and 1% MEM nonessential amino acids solution (MEMneaa). At 4 days post infection, culture medium was harvested, cleared by slow-speed centrifugation and titrated using Vero-E6 cells (ATCC CRL-1586), grown in DMEM supplemented with GlutaMAX, 3% FBS, 1% Pen/Strep and 1% Fungizone (DMEM +) at 37 °C and 5% CO_2_. Titers were determined using the Spearman-Kärber algorithm^22,23^.

### Mosquito rearing and feeding on lambs

Rockefeller strain *Ae. aegypti* mosquitoes (Bayer AG, Monheim, Germany) were maintained at Wageningen University, Wageningen, the Netherlands, as described^24^. Briefly, mosquitoes were kept in Bugdorm-1 rearing cages at a temperature of 27 °C with a 12:12 light:dark cycle and a relative humidity of 70% with a 6% glucose solution provided ad libitum. Mosquitoes were subsequently transported to biosafety level three (BSL-3) facilities of Wageningen Bioveterinary Research (Lelystad, the Netherlands), where the mosquitoes were maintained with sugar water (6% sucrose in H_2_O), provided via soaked cotton pads covered with a lid to prevent evaporation in an insect incubator (KBWF 240, Binder) at 28 °C at a humidity of 70% and a 16:8 light:dark cycle.

Mosquito feeding on lambs was preceded by sedating the lambs with IV administration of medetomidine (Sedator). When fully sedated, cardboard boxes containing 40–50 female mosquitoes were placed on the shaved inner thigh of each hind leg (Fig. 1b,c). After 20 min of feeding, cardboard boxes were removed and atipamezol (Atipam) was administered via intramuscular (IM) route to wake up the animals. Fully engorged mosquitoes were collected using an automated insect aspirator and maintained with sugar water (6% sucrose in H_2_O), provided via soaked cotton pads covered with a lid to prevent evaporation, in an insect incubator (KBWF 240, Binder) at 28 °C at a humidity of 70% and a 16:8 light:dark cycle.

### Feeding of mosquitoes using a Hemotek system

Blood meals to be used for Hemotek membrane feeding were prepared essentially as described before^25^. Briefly, erythrocytes were harvested from freshly collected bovine EDTA blood by slow-speed centrifugation (650 *xg*), followed by three wash steps with PBS. Washed erythrocytes were resuspended in L15 complete medium (L15 + 10% FBS, 2% TPB, 1% MEMneaa) to a concentration that is four times higher than found in blood. To prepare a blood meal, one part of the erythrocyte suspension was mixed with two parts of culture medium containing RVFV resulting in a final titer of 10^7.5^ TCID50/ml as determined on Vero-E6 cells.

Mosquitoes were allowed to take a RVFV-spiked blood meal through a Parafilm M membrane using the Hemotek PS5 feeding system (Discovery Workshops, Lancashire, United Kingdom). Feeding was performed in plastic buckets (1 l) covered with mosquito netting. After blood feeding for approximately 1.5–2 h, fully engorged mosquitoes were collected using an automated insect aspirator and maintained with sugar water (6% sucrose in H_2_O), provided via soaked cotton pads covered with a lid to prevent evaporation in an insect incubator (KBWF 240, Binder) at 28 °C at a humidity of 70% and a 16:8 light:dark cycle.

### Virus isolation

Virus isolation from plasma samples was performed using BHK-21 cells, seeded at a density of 20,000 cells/well in 96-wells plates. Serial dilutions of samples were incubated with the cells for 1.5 h before medium replacement. Cytopathic effect was evaluated after 5–7 days post infection. Virus titers (TCID_50_/ml) were determined using the Spearman-Kärber algorithm^22,23^.

To check for positive saliva, mosquitoes were sedated on a semi-permeable CO_2_-pad connected to 100% CO_2_ and wings and legs were removed. Saliva was collected by forced salivation using 20 µl filter tips containing 7 µl of a 1:1 mixture of FBS and 50% sucrose (capillary tube method). After 1–1.5 h, saliva samples were collected and used to inoculate Vero-E6 cell monolayers. Cytopathic effect (CPE) was scored 5–7 days later.

### Serology

Weekly collected serum samples were used to detect RVFV-specific antibodies using the ID Screen Rift Valley Fever Competition Multi-species ELISA (ID-VET). This ELISA measures percentage competition between antibodies present in test sera and a monoclonal antibody. Neutralizing antibodies were detected using the RVFV-4 s-based virus neutralization test as described^26^.

### RT-qPCR

Viral RNA was isolated with the NucliSENS easyMAG system according the manufacturer’s instructions (bioMerieux, France) from 0.5 ml plasma samples. Briefly, 5 µl RNA was used in a RVFV RT-qPCR using the LightCycler one-tube RNA Amplification Kit HybProbe (Roche, Almere, The Netherlands) in combination with a LightCycler 480 real-time PCR system (Roche) and the RVS forward primers (AAAGGAACAATGGACTCTGGTCA), the RVAs (CACTTCTTACTACCATGTCCTCCAAT) reverse primer and a FAM-labelled probe RVP (AAAGCTTTGATATCTCTCAGTGCCCCAA). Primers and probes were earlier described by Drosten et al.^27^. Virus isolations were performed on RT-qPCR positive samples with a threshold above 10^5^ RNA copies/ml as this was previously shown to be a cut-off point below which no live virus can be isolated.

### Pathology and (immuno)histopathology

Liver samples were placed on ice during the necropsies and subsequently stored at − 80 °C until virus isolations and RT-qPCR Tissue samples for histology and IHC were collected, placed in 10% neutral buffered formalin, embedded into paraffin and prepared for H&E staining or IHC staining for RVFV antigen using the RVFV Gn-specific 4-D4 mAb as described^5^.

### Statistics

For statistical analysis, mosquito feeding and mosquito saliva positive rates per group were compared by fitting logistic regression mixed models where lamb or membrane were introduced as random effects. To compare viremia (based on virus isolation results) the area under the curve (AUC) representing the overall viremia during the infected period was calculated for each infected sheep. This AUC and peak of viremia was used for comparison between groups, which was done by fitting linear regression models.

Additionally we also assessed the variability observed between groups on the above mentioned variables (feeding and saliva positive rates, AUC and peak viremia). For these comparisons, data were first assessed for normality using the Shapiro–Wilk test. If data from all groups were normally distributed, the Bartlett's test of homogeneity of variance was used. If the data did not have a normal distribution, the Fligner-Killeen test was applied.

Survival of infected lambs (time to death) was compared between experiment groups using Kaplan–Meier survival analysis and the mortality rates were compared fitting a logistic regression model.

For all comparisons, the threshold for significance was p < 0.05, for analysis involving multiple group comparison a Bonferroni correction was applied. All statistical analysis were performed using the statistical software package R^28^. The package MESS^29^ was used to compute the AUC.

### Ethics statement

The animal experiment was conducted in accordance with European regulations (EU directive 2010/63/EU) and the Dutch Law on Animal Experiments (Wod, ID number BWBR0003081). Permissions were granted by the Dutch Central Authority for Scientific Procedures on Animals (Permit Number: AVD4010020185564). All experimental protocols were approved by the Animal Ethics Committees of Wageningen Research.

## Supplementary Information

Supplementary Information
