# Supplementary material for: Reproducing the Rift Valley fever virus mosquito-lamb-mosquito transmission cycle
Source: Sci Rep. 2021 Jan 14;11:1477. doi: 10.1038/s41598-020-79267-1 (PMC7809480; doi:10.1038/s41598-020-79267-1)
Supplement: Supplementary file 1 — Supplementary Information [file 41598_2020_79267_MOESM1_ESM.pdf]

Supplementary Information

Reproducing the Rift Valley fever virus mosquito-lamb-mosquito transmission cycle

Paul J Wichgers Schreur [1], Rianka P. M. Vloet [1], Jet Kant [1], Lucien van Keulen [1], Jose L. Gonzales [1], Tessa M. Visser [2], Constantianus J.M. Koenraadt [2], Chantal B. F. Vogels [2,ϕ], and Jeroen Kortekaas [1,3*]

[1] Wageningen Bioveterinary Research, Lelystad, The Netherlands

[2] Laboratory of Entomology, Wageningen University & Research, Wageningen, The Netherlands.

[3] Laboratory of Virology, Wageningen University & Research, Wageningen, The Netherlands

[ϕ] Present address: Department of Epidemiology of Microbial Diseases, Yale School of Public Health, New Haven, CT, USA

*****Correspondence: jeroen.kortekaas@wur.nl; Tel.: +31 6 20919110

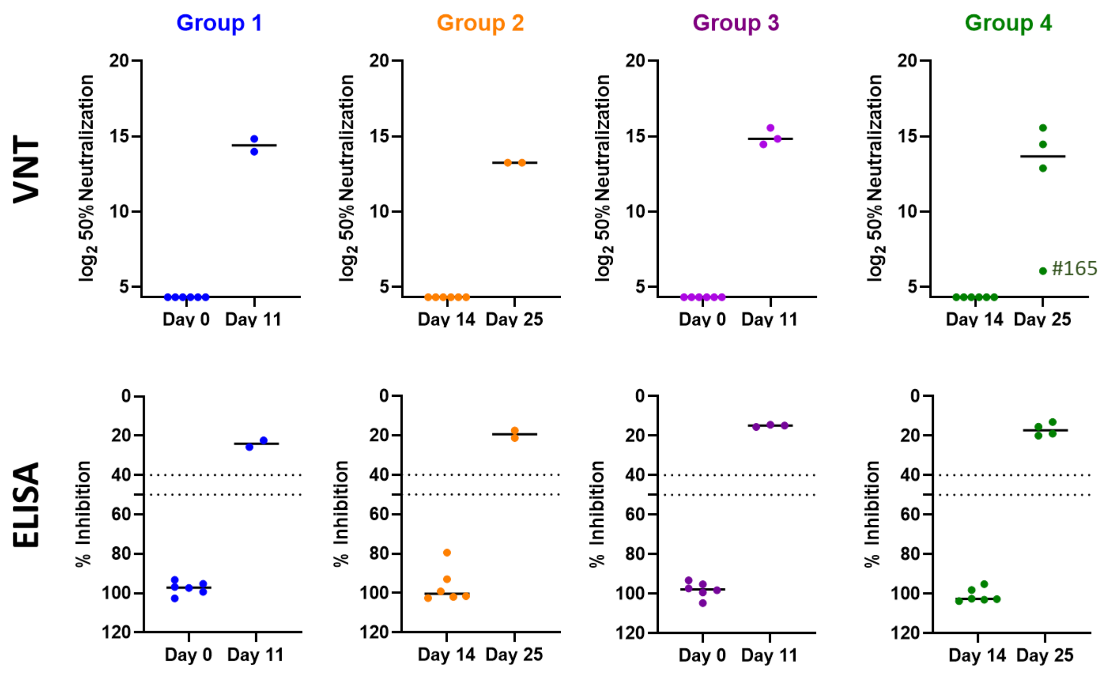

Supplementary Figure S1. Antibody responses after RVFV infection following needle inoculation or exposure to RVFV-positive mosquitoes. Sera collected from lambs before IV inoculation or mosquito exposure (day 0 for Groups 1 and 3; day 14 for Groups 2 and 4) and 11 days after infection via needle inoculation (Group 1) or mosquito exposure (Groups 2, 3, 4) were analyzed for the presence of anti-nucleocapsid (N) antibodies by the ID Screen® Rift Valley Fever Competition Multi-species ELISA (ID-VET) and neutralizing antibodies by virus-neutralization test[26].

Supplementary Table S1: Experimental details

Animals	
Species	Sheep
Breed and/or strain	Texel cross-breed
Source	Conventional sheep farm Netherlands
Sex	Male and female
Developmental stage	Between 8-16 weeks of age
Health status	Healthy upon enrolment
Weight	12-30 kg
Identification	By ear tag and by non-irritating coloured spray on the back of the animal
**Housing and Husbandry**	
Type of facility	BSL-3 facility
Type of housing	Stables (18 m^2)
Bedding material	Wood shavings
Number of animals per stable	12
Light/dark cycle	12/12
Temperature	20°C
Quality of water	Tap water, quality checked daily
Type of food	Hay, grass pellets and sheep grain
Access to water and food	*Ad libitum*
Access to food	Once per day
Environmental enrichment	None
Acclimatization time	7 days
**Experimental Procedure**	
Number experimental groups	4
Number of animals per group	6
Randomisation procedure	Animals were randomly allocated to groups by a veterinarian.
Experimental unit	Group
Route of administration	Intramuscular injection in jugular vein or exposure to mosquitoes in cardboard containers.
Anaesthesia and analgesia	Sedator and atipamezol
Method of euthanasia	Intravenous injection with sodium pentobarbital.
Humane endpoints	-The animal is recumbent and does not rise even after stimulation<br>-The animal is unable to drink<br>-The animal is lethargic (listless, apathic, non-responsive to stimuli)
Observations	The animals were observed for clinical signs once per day before, and twice per day after inoculation or mosquito exposure.

Supplementary Table S2. Mean and peak viremia measured in RVFV infected lambs[a]

Group	Area Under the Curve (AUC)		Peak viremia	
	Mean	SD	Mean	SD
1	6.28	0.52	6.21	0.53
2	6.41	0.30	6.33	0.32
3	5.53	1.40	5.47	1.52
4	5.71	1.97	5.59	2.02

[a]Numbers represent $TCID_{50}$/ml.
